# Electrocardiographic Markers of Adverse Left Ventricular Remodeling and Myocardial Fibrosis in Severe Aortic Stenosis

**DOI:** 10.3390/jcm12175588

**Published:** 2023-08-27

**Authors:** Giedrė Balčiūnaitė, Ieva Rudinskaitė, Darius Palionis, Justinas Besusparis, Edvardas Žurauskas, Vilius Janušauskas, Aleksejus Zorinas, Nomeda Valevičienė, Kęstutis Ručinskas, Peter Sogaard, Sigita Glaveckaitė

**Affiliations:** 1 Clinic of Cardiovascular Diseases, Institute of Clinical Medicine, Faculty of Medicine, Vilnius University, LT-08661 Vilnius, Lithuania; vilius.janusauskas@santa.lt (V.J.); aleksejus.zorinas@santa.lt (A.Z.); kestutis.rucinskas@santa.lt (K.R.); sigita.glaveckaite@santa.lt (S.G.); 2 Faculty of Medicine, Vilnius University, LT-03101 Vilnius, Lithuania; ievarudinskaite@gmail.com; 3 Department of Radiology, Nuclear Medicine and Medical Physics, Institute of Biomedical Sciences, Faculty of Medicine, Vilnius University, LT-08661 Vilnius, Lithuania; darius.palionis@santa.lt (D.P.); nomeda.valeviciene@santa.lt (N.V.); 4 Department of Pathology, Forensic Medicine and Pharmacology, Institute of Biomedical Sciences, Faculty of Medicine, Vilnius University, LT-08406 Vilnius, Lithuania; justinas.besusparis@vpc.lt (J.B.);; 5 Clinical Institute of Aalborg University, Aalborg University Hospital, Hobrovej 18-22, 9100 Aalborg, Denmark

**Keywords:** aortic stenosis, cardiovascular magnetic resonance, electrocardiographic strain, myocardial fibrosis, T1 mapping

## Abstract

The optimal timing for aortic valve replacement (AVR) in aortic stenosis (AS) is still controversial and may be guided by markers of adverse left ventricular (LV) remodeling. We aim to assess electrocardiographic (ECG) strain in relation to LV remodeling and myocardial fibrosis. 83 severe AS patients underwent surgical AVR, with preoperative 12-lead ECG, cardiovascular magnetic resonance with T1 mapping and echocardiography with global longitudinal strain analysis. Collagen volume fraction (CVF) was measured in myocardial biopsies sampled during AVR. Patients with ECG strain had more severe AS, more advanced LV remodeling and evidence of heart failure. Patients with ECG strain had more diffuse fibrosis, as evident by higher mean native T1 values (974.8 ± 34 ms vs. 946.5 ± 28 ms, *p* < 0.001). ECG strain was the only predictor of increased LV mass index on multivariate regression analysis (OR = 7.10, 95% CI 1.46–34.48, *p* = 0.02). Patients with persistent ECG strain at 1 year following AVR had more advanced LV remodeling and more histological fibrosis (CVF 12.5% vs. 7.3%, *p* = 0.009) at baseline assessment. Therefore, ECG strain is a marker of adverse LV remodeling and interstitial myocardial fibrosis. Lack of improvement in ECG strain following AVR indicates more advanced baseline LV injury and higher levels of myocardial fibrosis.

## 1. Introduction

Aortic stenosis (AS) is characterized by progressive valve narrowing that eventually leads to aortic valve (AV) intervention in developed countries [1,2]. Chronic left ventricular (LV) pressure overload results in progressive cardiac remodeling—adaptive LV hypertrophy initially, followed by myocyte degeneration and myocardial fibrosis [2,3,4]. These changes in myocardial structure ultimately result in increased myocardial stiffness and LV diastolic and systolic dysfunction, driving the transition from adaptive response to cardiac decompensation [5,6]. The extent of LV damage is a powerful predictor of postoperative clinical outcomes [7], and both LV dysfunction and myocardial fibrosis have been associated with a poor prognosis following aortic valve replacement (AVR) [8,9,10,11,12]. The optimal timing of AVR is still being debated and is currently based on the onset of AS-related symptoms and/or evidence of LV dysfunction [13]. Therefore, there is increasing clinical interest in assessing myocardial interstitium as a possible marker of cardiac decompensation in the optimal management of AS patients.

Myocardial biopsy, followed by histological analysis, is considered the gold standard for the assessment of myocardial fibrosis [14]; however, its invasive nature may be unsuitable in frail elderly AS patients. Cardiovascular magnetic resonance (CMR) provides a non-invasive and global assessment of myocardial fibrosis: diffuse interstitial expansion is measured by T1 mapping [15], while focal fibrosis is quantified by the late gadolinium enhancement (LGE) technique [16,17]. Although CMR is an excellent tool to assess myocardial fibrosis, its cost and restricted availability limit widespread use in everyday clinical practice. CMR may also be ineligible in patients having contraindications to the scan or contrast media or difficulties holding their breath. On the contrary, an electrocardiogram (ECG) is a safe and widespread tool to quickly assess markers indicating pathological LV remodeling.

ST segment down-sloping and T-wave inversions on the ECG are attributed to myocardial ischemia (18). However, these ECG abnormalities may be present in the absence of coronary artery disease (CAD). The increase in LV mass also prolongs and distorts electrical impulse propagation within the myocardium, creating depolarization abnormalities. LV strain pattern of lateral ST depression and T-wave inversion (or ECG strain) and increased QRS voltage are the most acknowledged ECG markers of LV hypertrophy in AS [8,10,11,18,19,20]. ECG strain has been associated with an increased risk of cardiovascular mortality and morbidity in both asymptomatic and symptomatic severe AS patients [21,22]. The implied underlying pathophysiology of ECG strain is an oxygen demand-supply imbalance in the hypertrophied myocardium; thus, it is considered a sign of subendocardial ischemia and fibrosis [18]. Although the link between LV hypertrophy and ECG strain is well established, the link between ECG alterations and myocardial fibrosis has not been well investigated.

Our prospective study aims to (i) assess associations between ECG strain and LV remodeling by integrating echocardiographic and CMR data, (ii) analyze the link between ECG strain and invasively and non-invasively measured myocardial fibrosis, and (iii) investigate whether ECG evidence of LV remodeling will resolve following AVR.

## 2. Materials and Methods

### 2.1. Study Design and Population

This is a prospective observational study carried out at Vilnius University Hospital between November 2018 and December 2020. The study included patients with severe symptomatic AS who were scheduled for surgical AVR according to the current treatment recommendations [13]. The study was approved by the local biomedical research ethics committee (number: 158200-18/9-1014-558) and conformed to the principles of the Helsinki Declaration. Patients were recruited prior to a pre-operative assessment and underwent a clinical assessment that included clinical history, the Minnesota Living with Heart Failure Questionnaire, the 6-min walking test, blood sampling [for hematocrit, renal function, brain natriuretic peptide (BNP) and high sensitivity troponin I (Hs-Tn-I)], a transthoracic echocardiogram, and CMR. The study data were collected and stored in an online database, REDCap (Research Electronic Data Capture) [23].

### 2.2. Inclusion/Exclusion Criteria

**Figure 1:**
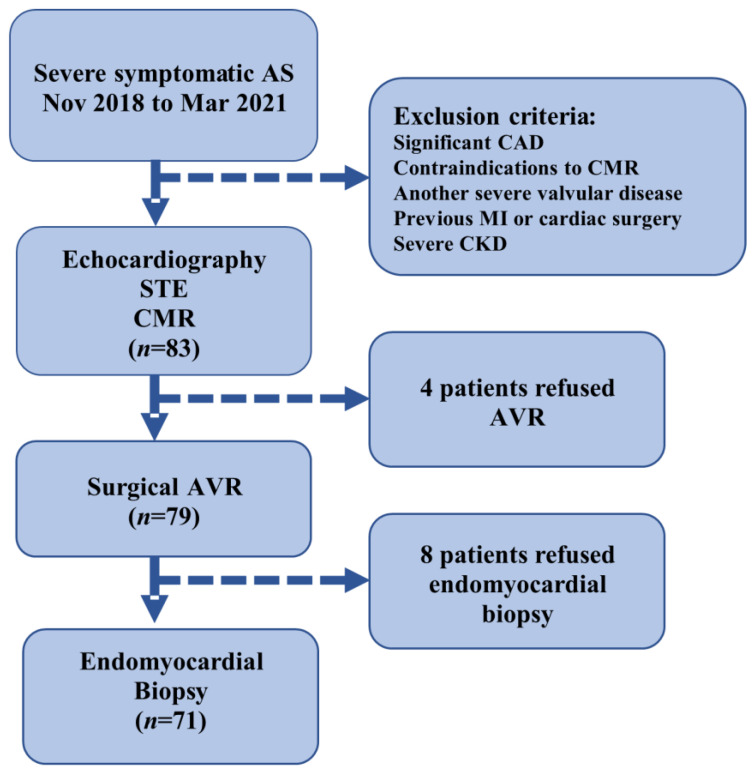
FIB-AS study flow chart.

The inclusion criteria were patients who were undergoing AVR for severe AS [defined as aortic valve area (AVA) ≤ 1 cm^2^ or AVA index ≤ 0.6 cm^2^/m^2^, as determined by echocardiography], age > 18 years, and ability to undergo a CMR scan and to sign consent to the study protocol. The exclusion criteria were severe valve disease other than AS, significant CAD (>50% lesion in any epicardial coronary artery), history of myocardial infarction, estimated glomerular filtration rate <30 mL/min/1.73 m^2^, CMR-incompatible devices, persistent atrial tachyarrhythmias, and previous cardiac surgery. The main reasons for non-eligibility were significant CAD, renal dysfunction, and other valvular abnormalities. Of the 83 participants, 79 underwent surgical AVR and 4 postponed surgeries due to the COVID-19 epidemiologic situation. (Figure 1).

### 2.3. Cardiac Imaging

#### 2.3.1. ECG Analysis

A standard 12-lead ECG was obtained from all patients preoperatively, 3 and 12 months after surgical AVR. The following automatically provided ECG parameters were collected: heart rate, PQ and QRS duration. The QRS voltage and Sokolow-Lyon index (S-L) were calculated manually. S-L index is calculated as the sum of amplitudes of the S wave in lead V1 and the R wave in lead V5 or V6 based on the highest R wave amplitude [24]. LV strain on ECG was defined as ≥1 mm ST-segment depression measured from the J point with asymmetrical T wave inversion in the lateral leads (I, aVL, V5, and V6) [25]. ECG interpretation and the presence of ECG strain were determined by two independent investigators who were blinded to the clinical data and cardiac imaging findings.

#### 2.3.2. Echocardiography

Transthoracic two-dimensional echocardiography was performed using a commercially available Vivid ultrasound system (S70, E9, or E95) (GE Healthcare, Horten, Norway), and the data were stored on a dedicated workstation for subsequent offline analysis. LV systolic and diastolic function were evaluated according to echocardiographic guidelines, and AVA was calculated using the continuity equation [26,27]. From the 2D grey-scale images of the apical 2-, 3-, and 4-chamber views, LV global longitudinal strain (GLS) was measured and processed off-line using commercially available software (EchoPac 112.0.1, GE Medical Systems, Horten, Norway) [28]. The frame rate was adjusted to 50 to 80 frames/s. End-systole was defined based on the closure click on the spectral tracing of the pulsed-wave Doppler of AV flow. GLS was acquired using the average regional strain curves 16-segment model for 2D speckle tracking echocardiography. Segments with poor quality tracking or aberrant curves (despite manual adjustment) were removed from the analysis. Due to missing data or poor image quality, strain analysis was completed for 77 of 83 patients.

#### 2.3.3. CMR Protocol

CMR scans were obtained using standard protocols on a 1.5 T Siemens Aera scanner with surface coils and prospective ECG triggering. LV end-systolic and end-diastolic diameters and maximum wall thickness were traced and recorded from the short-axis and long-axis views of the standard ECG-gated steady-state-free precession cine sequence. LV volumes, mass, and ejection fraction were measured using commercial software (suiteHEART^®^, Version 5.0.1) from a stack of sequential 8-mm short-axis slices (0–2-mm gap) from the atrioventricular ring to the apex. Measurements were indexed to body surface area in m² (using the DuBois formula). To detect delayed hyperenhancement, images were acquired 10–15 min after intravenous administration of gadobutrol (0.2 mmol/kg) (Gadovist, Bayer AG, Leverkusen, Germany) using a breath-hold segmented inversion recovery fast-gradient echo sequence in the short-axis and long-axis planes of the LV, with an 8-mm slice thickness and 20% distance factor. The region of myocardial fibrosis was defined as the sum of pixels with signal intensity above 5 standard deviations of the normal remote myocardium in each short-axis slice. The presence of LGE was determined qualitatively by two independent readers who were blinded to the clinical data. T1 mapping images were acquired in 4-chamber long-axis and short-axis images (at the midventricular levels) before and 15 min after contrast administration. All T1 mapping images were acquired using the modified Look-Locker inversion-recovery sequence [29,30] with the Motion Correction technique. T1 maps were generated from the CMR workstation after in-line motion correction just after image acquisition. Regions of interest were drawn manually in the blood and septum at the midventricular level on the short-axis image, excluding the myocardium with LGE. The ECV of the myocardium was calculated as follows: ECV% = (ΔR1m/ΔR1b) × (1 − hematocrit level) × 100, where R1 is 1/T1, R1m is R1 in the myocardium, R1b is R1 in the blood, and ΔR1 is the change in relaxation [31]. Due to incomplete datasets, T1 mapping parameters were measured in 67 of 83 patients.

### 2.4. Histological Analysis

Biopsy specimens for histological analysis were taken at the time of surgical AVR. The samples were obtained under direct supervision by the surgical team using a surgical scalpel from the basal anteroseptal just after the removal of the diseased AV. One intraoperative myocardial biopsy sample (mean area 22.5 ± 12 mm^2^) was taken from each patient. All myocardial tissue samples were fixed in 10% neutral buffered formalin and embedded in paraffin. Sections (3 µm thick) were sliced on a Leica RM2145 microtome and stained with hematoxylin and eosin and Masson‘s trichrome. Digital images were captured by an Aperio Scan-Scope XT Slide Scanner (Aperio Technologies, Vista, CA, USA) under 20× objective magnification (0.5 µm resolution). All biopsy samples were examined by histologists who were blinded to the clinical and CMR data. The fraction of myocardial volume that was occupied by collagen tissue (collagen volume fraction, CVF) was determined by quantitative morphometry on an automated image analysis system (PIXEL^TM^). The area of myocardial fibrosis was calculated using the PIXEL^TM^ Area Quantification v2.1.11 algorithm (IndicaLabs, Albuquerque, NM, USA) [32]. The subendocardial layer was defined as 1 mm from the endocardial surface, whereas the rest of the tissue sample was defined as the midmyocardial layer.

### 2.5. Statistical Analysis

Continuous variables were presented as mean ± standard deviation or median (interquartile range). Categorical variables were recorded as frequencies (percentages). A two-sample student’s *t*-test was used to compare normally distributed variable means between two groups, while the Mann–Whitney U test was employed for skewed data. The chi-squared (χ^2^) test or Fisher’s exact test was used to identify statistically significant differences for categorical variables. Pearson’s and Spearman’s correlation coefficients were used to estimate the relationships between continuous variables. Univariate and multivariate logistic regression analysis was performed. Results from the logistic regression analysis were presented as odds ratios (ORs) and 95% confidence intervals (CIs). A 2-sided value of *p* < 0.05 was considered statistically significant. The SPSS statistical software (IBM SPSS 28.0.1) was used for data analysis.

## 3. Results

A total of 83 patients with severe AS were enrolled in the study. The mean age of the study subjects was 66.5 ± 8.6 years (range: 45–84 years), and 58% were females. The vast majority of patients had high-gradient severe AS: the median AVA index was 0.45 cm^2^/m^2^ (0.35–0.53), and the median mean transvalvular gradient was 54.9 mm Hg (44.4–70). Further, 89% of patients had preserved LV ejection fraction (LVEF): mean LVEF 66.7 ± 12.8%. Out of 83 patients with severe AS, 43.4% had the strain pattern on their ECGs at baseline assessment. Patients were divided into two groups according to the presence of ECG strain. Both groups were balanced in terms of their age and comorbidities. Patients with ECG strain were more frequently male (*p* = 0.002) and had lower systolic (*p* < 0.001) and diastolic blood pressures (*p* < 0.001). When ECG parameters were examined, LV hypertrophy by Sokolow-Lyon criterion was more prevalent (63.2% vs. 25.0%, *p* < 0.001); QRS voltage (*p* < 0.001) and QRS duration (*p* = 0.016) were greater in the group with ECG strain. Patients with ECG strain also had several times higher BNP (*p* < 0.001) and Hs-Tn-I (*p* < 0.001) levels. The baseline clinical characteristics of the patients according to the presence of ECG strain are summarized in Table 1.

### 3.1. ECG Strain and LV Remodeling

Comparative analysis revealed that patients with ECG strain had more advanced AS, as evident by the higher peak AV velocity (*p* = 0.008) and higher mean transvalvular gradient (*p* = 0.003). Patients with strain pattern on ECG had more advanced LV remodeling: significantly thicker LV walls (*p* < 0.001), larger LV dimensions (*p* < 0.001), greater LV mass index (*p* < 0.001), and larger LV end-systolic (*p* < 0.001) and end-diastolic (*p* < 0.001) volume indexes. This group of patients also showed signs of more advanced diastolic dysfunction and elevated LV filling pressures, as evident by higher septal E/e’ (*p* = 0.011) and larger left atrial volume index (*p* < 0.001). Furthermore, these patients showed worse LV systolic function, as they had lower GLS (*p* < 0.001), lower LVEF (*p* < 0.001), and higher prevalence of reduced LVEF (*p* = 0.009). The imaging characteristics of the patients according to the presence of ECG strain are summarized in Table 2.

### 3.2. ECG Strain and Myocardial Fibrosis

**Figure 2:**
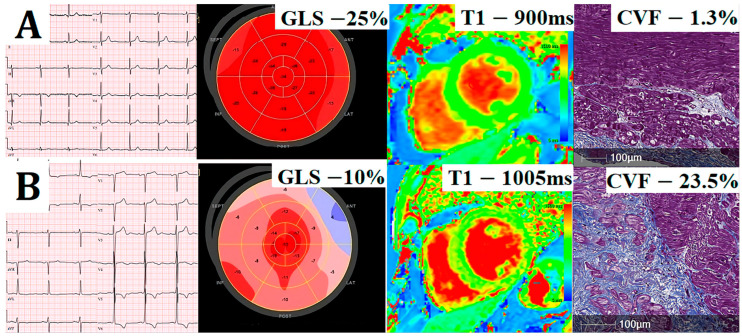
Illustrative comparison of cardiovascular imaging and histology data of two exemplar patients: electrocardiography (Column 1), global longitudinal strain (GLS; Column 2), matching native T1 (Column 3), and collagen volume fraction (CVF) in myocardial biopsies stained with Masson’s trichrome (Column 4). Patient without ECG changes (**A**) has preserved GLS, low native T1, and low histological fibrosis (CVF of 1.3%), whereas patient with ECG strain (**B**) has significantly reduced GLS, high native T1, and extensive histological fibrosis (CVF 23.5%).

The mean native T1 was 959.6 ± 33.7 ms (range: 897–1044 ms), and the median ECV was 23.1% (20.8–24.9) (range: 15.7–34.4%). We found that patients with ECG strain had higher native T1 (*p* < 0.001); however, no significant differences in the mean ECV values were noted between the study groups (*p* = 0.821). Focal myocardial fibrosis detected by LGE-CMR was present in 74% of patients. There was a tendency for higher prevalence of LGE in patients with ECG strain; however, it did not reach statistical significance (83% vs. 66%, *p* = 0.075). In regard to histological analysis, we found no significant differences in histologically measured myocardial fibrosis (mean CVF values) between the patient groups with and without ECG strain (16.6 ± 10.2% vs. 15.7 ± 8.7%, *p* = 0.679). Representative images of patients with and without ECG strain are shown in Figure 2.

### 3.3. Analysis of Associations

**Figure 3:**
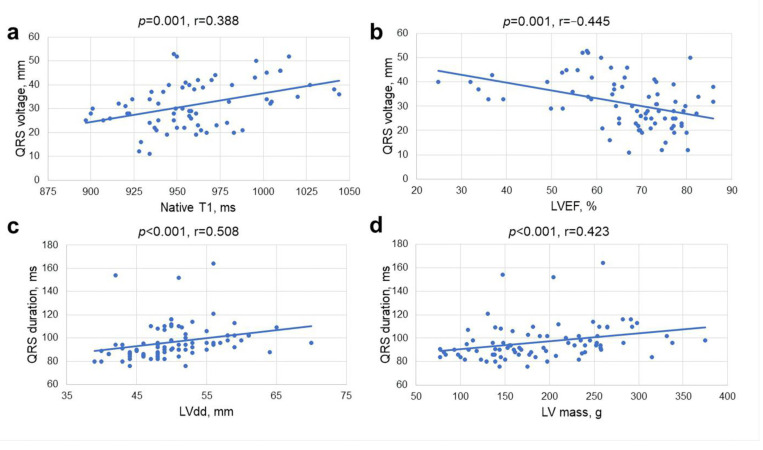
Correlations between QRS voltage and native T1 (**a**), LV ejection fraction (LVEF) (**b**), QRS duration and LV diastolic diameter (CMR) (**c**), and LV mass (CMR) (**d**).

We observed significant correlations between QRS duration and QRS voltage and imaging parameters of LV remodeling: LV end-diastolic diameter (r = 0.508, *p* < 0.001 and r = 0.220, *p* = 0.046, respectively), LV end-systolic diameter (r = 0.439, *p* < 0.001 and r = 0.371, *p* = 0.001, respectively), LV end-diastolic (r = 0.364, *p* = 0.001 and r = 0.549, *p* < 0.001, respectively) and LV end-systolic volume indexes (r = 0.337, *p* = 0.002 and r = 0.552, *p* < 0.001, respectively), and LV mass index (r = 0.389, *p* < 0.001 and r = 0.525, *p* < 0.001, respectively). QRS duration and QRS voltage negatively correlated with parameters of LV systolic function: LVEF (r = −0.230, *p* = 0.037 and r = −0.445, *p* < 0.001, respectively) and GLS (r = −0.301, *p* = 0.008 and r = −0.381, *p* = 0.001, respectively) (Figure 3). QRS duration weakly but significantly correlated with parameters related to LV diastolic dysfunction and elevated filling pressures: E wave deceleration time (r = −0.246, *p* = 0.035), left atrial volume index (r = 0.246, *p* = 0.027) and estimated pulmonary artery systolic pressure (r = 0.369, *p* = 0.021). In regard to serum biomarkers, QRS duration and QRS voltage also correlated with Tn-I (r = 0.367, *p* = 0.001 and r = 0.344, *p* = 0.002, respectively) and BNP (r = 0.251, *p* = 0.023 and r = 0.438, *p* < 0.001, respectively). When analyzing associations between ECG and CMR parametric mapping data, QRS voltage correlated with native T1 (r = 0.388, *p* = 0.001). No correlations were found between selected ECG variables (QRS duration and QRS voltage) and histological myocardial fibrosis (CVF) or ECV.

### 3.4. ECG Parameters as Independent Prognostic Factors

The univariate logistic regression analysis revealed that male sex, higher levels of BNP, increased QRS voltage, lower LVEF, reduced GLS, and ECG strain were statistically significant predictors for increased LV mass index (Table 3). Only ECG strain remained a significant predictor of increased LV mass index in a multivariate regression analysis. Analysis of the predictors of diffuse myocardial fibrosis revealed that ECG strain, increased QRS voltage, and reduced LVEF and GLS were predictive of increased native T1; however, no significant associations were noted on multivariate regression analysis (Table 3).

### 3.5. ECG Changes at Follow-Up

The data of 76 and 59 patients were available at 3- and 12-month follow-up visits, respectively. ECG parameters before and after surgical AVR are shown in Table 4. Comparative analysis of ECG parameters included postoperative ECGs with a new first-degree AV block (5 patients), left bundle branch block (8 patients), and right bundle branch block (2 patients). Two patients were excluded from further analysis due to cardiac pacemaker activity seen on the ECG. Our results demonstrated that QRS voltage had significantly decreased at 3 and 12 months after the surgery [30 mm (23–39) vs. 23 mm (18.5–27) vs. 19.5 mm (16–24), respectively, *p* < 0.001]. Furthermore, the prevalence of ECG strain gradually decreased from 43% to 17% in 1 year (*p* = 0.001). We also observed a significant increase in QRS duration at 3 months following AVR (*p* < 0.05); the result was likely related to newly developed intraventricular conduction abnormalities soon after the surgery (left and right bundle branch blocks).

**Figure 4:**
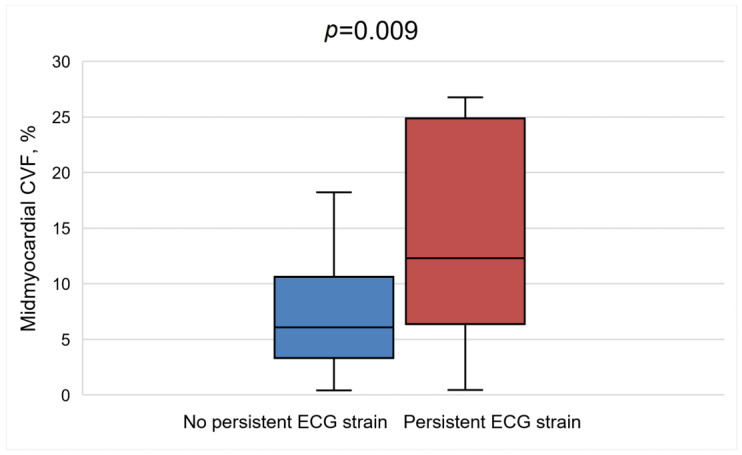
The graph shows a comparison of histological myocardial fibrosis between the patient’s group with and without ECG strain at 1 year following aortic valve replacement. A higher proportion of collagen volume fraction (CVF) at baseline assessment was detected in patients with persistent ECG strain compared to patients with no evidence of ECG strain.

Further analysis revealed that patients with persistent ECG strain at 1 year following AVR had lower systolic and diastolic blood pressures (*p* = 0.017 and *p* = 0.040, respectively), greater QRS duration [102 ms (94–106.3) vs. 92 ms (86–101), *p* = 0.042], and more advanced heart failure, as evident by higher levels of BNP (*p* = 0.005) at baseline. These patients also had more advanced baseline LV remodeling, as they had greater LV mass (*p* = 0.023) and larger indexed LV end-systolic (*p* = 0.003) and LV end-diastolic (*p* = 0.010) volumes (Table 5). Furthermore, this group of patients showed worse baseline LV and right ventricle systolic functions, as they had significantly reduced GLS (*p* < 0.001), lower LVEF (*p* < 0.001), and lower right ventricle ejection fraction (*p* < 0.002). In regard to histological analysis, patients with persistent ECG strain had significantly more fibrosis in the midmyocardial layer on histological analysis (12.5 ± 9.9% vs. 7.3 ± 4.7%, *p* = 0.009) (Figure 4).

## 4. Discussion

This is a prospective study integrating data of multimodality cardiac imaging and ECG changes in conjunction with a histological analysis in patients with severe AS undergoing surgical AVR. The main study findings are as follows: (i) the presence of LV strain pattern on ECG is associated with adverse LV remodeling and higher native T1 values representing interstitial expansion due to fibrosis, (ii) the presence of LV strain pattern on ECG is an independent predictor of increased LV mass, and (iii) persistence of LV strain pattern on ECG at 1 year following AVR represents more advanced LV remodeling with higher degree of histological fibrosis at baseline.

### 4.1. ECG Strain and LV Remodeling

It is of great importance to accurately assess cardiac damage in AS, as it is the main determinant of postoperative clinical outcomes. Structural LV myocardial changes and myocardial fibrosis in AS are associated with electrical alterations and may affect both myocardial depolarization and repolarization [33]. LV strain pattern on ECG has been described in various proportions of patients with AS, and the incidence rises with the severity of the disease [34] and is higher in patients with reduced LVEF [35]. The ECG strain pattern was frequent in our cohort, found in 43% of patients, which was higher than previously reported (21–39%) [22,36,37]. Although its pathophysiology remains debated, previous studies showed that ECG strain is associated with an advanced hypertrophic response to pressure overload [21]. There is considerable interest in early, objective, and easily determined markers of LV injury that could guide treatment options and identify patients who would benefit from early AVR. ECG strain, in conjunction with other clinical and imaging data, could guide decision-making, as it reflects adverse LV remodeling. Patients with ECG strain had larger LV mass and volumes and evidence of LV systolic and diastolic dysfunction. Patients with strain patterns on ECG also had more severe AV stenosis, as evident by the higher transvalvular gradient. Moreover, these patients had higher levels of serum biomarkers indicative of heart failure and myocardial injury. Therefore, the detection of ECG strain in AS patients should alert clinicians to proceed to a more detailed LV assessment and avoid management delays. This may be particularly useful in deciding in favor of early surgery in asymptomatic severe AS patients or borderline cases.

### 4.2. ECG Strain and Myocardial Fibrosis

ECG strain has been identified as an explicit electrocardiographic marker of midwall LV myocardial fibrosis as a result of subendocardial ischemia. In a study of 102 patients with mild to severe AS, ECG strain was predictive of diffuse and focal myocardial fibrosis assessed by CMR [19]. Our study adds to these data, demonstrating a close association between ECG strain and myocardial fibrosis. We found that patients with ECG strain had significantly higher degrees of diffuse myocardial fibrosis, as evidenced by higher native T1 values. Therefore, ECG strain not only reflects an increase in LV mass due to cellular growth but is also a marker of an increase in interstitial fibrosis. In our cohort, patients with ECG strain also had a higher prevalence of LGE when compared to patients without ECG strain (86% vs. 66%), indicating more advanced and irreversible myocardial injury. Interestingly, 66% of patients with no ECG strain pattern had evidence of focal fibrosis on LGE-CMR. It is known that fibrotic tissue is electrically inert and may reduce the ECG voltage and can mask the ECG changes of increased LV mass, which can explain the limited sensitivity of the ECG for detecting increased LV mass in some patients [38]. However, this probably does not apply to our cohort of patients, as although the replacement fibrosis was quite prevalent, it was not extensive, as usually only 1 or 2 LV segments per patient were affected. The strength of the current study is the exclusion of patients with obstructive CAD. Therefore, the detected fibrotic changes in the LV myocardium in our study are attributed solely to the presence of the valvular lesion. Our findings support the role of oxygen supply-demand mismatch and secondary ischemia in the hypertrophied LV myocardium with the development of myocardial fibrosis in the absence of obstructive coronary lesions.

Long-standing pressure overload and changes in LV myocardium and collagen accumulation have negative effects on myocardial deformation. GLS is an early and sensitive marker of LV dysfunction and correlates with histological fibrosis [39,40]. We found significantly reduced longitudinal deformation in patients with ECG strain in comparison to patients without ECG strain (GLS −15% vs. −20%). It has been recently shown that a GLS threshold of −15.0% was associated with replacement myocardial fibrosis in AS patients and that GLS values above this threshold were predictive of adverse cardiovascular events [41]. Therefore, the presence of ECG strain in pre-operative assessment may help to identify patients at risk. Unexpectedly, we did not detect differences in histologically measured myocardial fibrosis (CVF) between the groups with and without ECG strain. It is likely that a larger sample size is required to demonstrate this association. A sampling error could be another possible explanation, as only one biopsy sample per patient was analyzed, and fibrotic changes may not be equally distributed throughout the ventricular wall. Further, histological analysis revealed regional myocardial changes, which may not correspond to the global electrical activity of the entire LV.

### 4.3. ECG Strain at Follow-Up

The reduction of LV mass within the first years after the AV surgery has been previously reported [26,42,43], and LV reverse remodeling is an important indicator related to long-term prognosis [44]. Our results are consistent with earlier studies, showing the regression of electrocardiographic markers of LV hypertrophy after the AVR [10,23]. We observed a gradual regression of QRS voltage already at 3 months, with continued regression at 1 year following AVR. The prevalence of ECG strain also decreased as soon as 3 months following AVR. However, ECG strain persisted in 17% of patients at 1 year, signaling an incomplete LV recovery. It has been shown that LV hypertrophy might partially persist in some patients, contributing to persistent LV dysfunction and lack of clinical improvement. This lack of improvement in ECG alterations reflects more advanced baseline myocardial damage that has accumulated during decades of progressive AV disease. Patients with persistent ECG strain in our cohort had more advanced LV remodeling on pre-operative assessment, with larger LV mass and volumes. These patients also showed evidence of advanced heart failure, as they had lower LVEF, severely reduced GLS, and significantly increased BNP levels. At the histological level, we found that a higher amount of fibrosis was also measured in the myocardium of these patients (CVF 12.5 vs. 7.3, *p* = 0.009). These findings suggest that surgery might have been performed too late for this group of patients and that LV structural and functional changes are only partially reversible or require more time to recover. Interestingly, a difference in the baseline histological fibrosis was detected in the midmyocardial but not the subendocardial layer. We have previously shown that various degrees of subendocardial fibrosis can be detected in biopsy samples of most AS patients and that the subendocardial region is affected by fibrosis the most [39], but the fibrosis extends to deeper myocardial layers and spreads to midmyocardium probably only in patients with more advanced disease. LV reverse remodeling and its impact on clinical outcomes have been investigated in a recent study of 132 patients with severe AS undergoing surgical AVR [45]. In that study, the presence of severe fibrosis at the time of surgery has been associated with less regression of LV hypertrophy and higher postoperative mortality, confirming its prognostic importance. The association between ECG strain and LV reverse remodeling in AS patients has also been investigated in a TAVI cohort. In a study of 207 severe AS patients referred for TAVI, patients with higher risk scores, combining age, sex, ECG strain, increased Hs-Tn-I, and peak AV velocity, had less LV reverse remodeling at 1 year [46]. These data indicate the importance of repeated LV assessment following AV intervention and additional management of patients with less LV improvement and persistent risk for adverse events.

### 4.4. Prognostic Value of ECG Strain

The clinical impact of pre-operative ECG markers of LV myocardial damage was explored in several recent studies. Coisne et al., in a large cohort of 1122 severe AS patients undergoing surgical AVR, showed that both ECG strain and conduction abnormalities were associated with major adverse cardiac events and all-cause and cardiovascular deaths [22]. The prognostic ability of ECG markers has also been demonstrated in TAVI cohorts. Al-Hijji et al. indicated that ECG strain is an independent predictor of long-term mortality post-TAVI [35]. Heger et al., in a study of 585 severe AS patients referred for TAVI, demonstrated that ECG strain was predictive of heart failure hospitalization [14]. Based on these results, ECG strain has been linked to excess cardiovascular morbidity and mortality in patients with AS. The association of ECG alterations with abnormalities of LV structure and function may, in part, explain the adverse prognosis associated with ECG strain. The two most common cardiovascular causes of death in the AS population are heart failure and sudden cardiac death [47]. Adverse LV remodeling and myocardial fibrosis can be linked to both causes, as they increase the likelihood of ventricular arrhythmias and cardiac decompensation. In our cohort, we did not obtain prognostic data due to the relatively small sample size and a short follow-up period. In summary, ECG may help to identify patients at risk and have clear advantages over other diagnostic tests, as it is a low-cost, widely accessible, easily interpretable, and non-invasive diagnostic tool. Patients with high-risk ECG features may benefit from further risk stratification with advanced cardiac imaging—speckle tracking echocardiography or CMR—depending on local expertise and availability of resources.

## 5. Conclusions

ECG strain is a marker of advanced structural and functional LV remodeling and interstitial myocardial fibrosis. ECG strain in a pre-operative assessment may help to identify patients at risk who may benefit from further advanced cardiac imaging and earlier intervention. Lack of improvement in ECG strain following AVR indicates a subgroup of patients with more advanced LV damage and higher levels of myocardial fibrosis who may require closer follow-up and additional medical management.

## 6. Limitations

This is a single-center study with a limited sample size, which reduces our statistical power. However, the size of our cohort is comparable to other studies on the same topic and includes a significant number of myocardial biopsies. Due to the COVID-19 pandemic, delays in patient examinations and surgeries were experienced, causing uneven time frames between the preoperative patient assessment (ECG, echocardiography, and CMR) and surgery with myocardial sampling, potentially affecting the final result. Furthermore, the proportion of histologically measured myocardial fibrosis could have been affected by the size and depth of biopsy samples, as more superficially sampled and smaller biopsies may contain a higher proportion of fibrotic tissue in comparison to larger biopsy samples. Extracellular volume fraction changes in AS patients are relatively small, and the detection of small deviations from normal ranges warrants a higher sample size. Lastly, we included only isolated AS patients undergoing surgical AVR; therefore, the results cannot be generalized to patients with concomitant CAD, mixed valvular lesions, or TAVI cohorts.

## Figures and Tables

**Table 1:** Baseline characteristics of the study cohort and patient groups stratified by the presence of the ECG strain.

Variable	All Patients (*n* = 83)	No ECG Strain (*n* = 47)	ECG Strain (*n* = 36)	*p*-Value
Age, yrs	66.5 ± 8.6	67.2 ± 8.3	65.4 ± 9.1	0.357
Male gender	35 (42.2)	13 (27.7)	22 (61.1)	**0.002**
BSA, m^2^	1.9 ± 0.2	1.9 ± 0.2	2 ± 0.2	0.165
Systolic BP, mm Hg	149.6 ± 24.8	157.7 ± 23.7	139.0 ± 22.3	**<0.001**
Diastolic BP, mm Hg	84.0 ± 11.7	88.4 ± 10.1	78.2 ± 11.2	**<0.001**
Comorbidities				
Hypertension	73 (88)	42 (89.4)	31 (86.1)	0.740
Dyslipidemia	67 (80.7)	40 (85.1)	27 (75)	0.247
Unobstructive CAD	39 (47)	20 (42.6)	19 (52.8)	0.355
Diabetes mellitus	15 (18.1)	10 (21.3)	5 (13.9)	0.389
Risk scores				
STS-PROM, %	1.6 (1.2–2.4)	1.8 (1.4–2.5)	1.3 (1.0–1.9)	**0.004**
EuroSCORE II, %	1 (0.7–1.6)	1.1 (0.8–1.6)	1 (0.7–1.7)	0.695
Functional status				
NYHA f. cl. *				
I–II	40 (48.2)	26 (55.3)	14 (38.9)	0.138 *
III–IV	43 (51.8)	21 (44.7)	22 (61.1)	
MLHFQ score	32.5 (18.5–52.8)	40.5 (19–56)	30 (17–40.8)	0.306
6 MWT, m	369 (300–420)	360 (294.8–420)	388.5 (322.5–420)	0.489
Blood tests				
eGFR, ml/min/1.73 m^2^	85 (69–90)	85 (69–90)	85 (67.5–90)	0.875
Hs-Tn-I, pg/L	9.1 (5–18.7)	6 (4–13)	15.5 (9–29)	**<0.001**
BNP, pg/L	130 (65.2–361.9)	80.2 (46.5–163.2)	297.2 (117.7–812.8)	**<0.001**
ECG parameters				
Heart rate, beats/min	75 (65–86)	75 (68–86)	75.5 (64.3–87.5)	0.890
PQ duration, ms	165 (153.5–180)	162 (150–176)	168 (160–184)	0.067
QRS duration, ms	94 (86–102)	90 (85–98)	96 (90.3–108.5)	**0.016**
S-L, mm	30.9 ± 9.9	25.3 ± 7.3	38.1 ± 8.1	**<0.001**
S-L ≥ 35 mm, %	28 (33.7)	6 (12.8)	22 (61.1)	**<0.001**

The boldface values indicate statistical significance. Continuous variables are presented as mean ± SD or median (IQR). Categorical variables are expressed as n (%). 6 MWT, 6-min walking test; BNP, brain natriuretic peptide; BP, blood pressure; BSA, body surface area; CAD, coronary artery disease; ECG, electrocardiography; eGFR, estimated glomerular filtration rate; EuroScore II, European System for Cardiac Operative Risk Evaluation II score; Hs-Tn-I, high-sensitivity troponin I; MLHFQ, Minnesota Living with Heart Failure Questionnaire; NYHA, New York Heart Association; S-L, Sokolow Lyon voltage criteria; STS, Society of Thoracic Surgeons’ risk model score. * *p*-value for comparison among NYHA I and II vs. III and IV.

**Table 2:** Cardiovascular imaging and histology data of the study cohort and patient groups stratified by the presence of ECG strain.

	All Patients (*n* = 83)	No ECG Strain (*n* = 47)	ECG Strain (*n* = 36)	*p*-Value
Echocardiography Data				
AVA, cm^2^	0.84 ± 0.2	0.85 ± 0.2	0.83 ± 0.2	0.612
AVA index, cm^2^/m^2^	0.45 (0.35–0.53)	0.47 (0.4–0.53)	0.41 (0.33–0.53)	0.230
Peak AV velocity, m/s	4.9 (4.4–5.3)	4.5 (4.2–5.2)	5.0 (4.7–5.5)	**0.008**
Mean AV gradient, mm Hg	54.9 (44.4–70.0)	49 (42.0–64.0)	60.5 (52.5–77.9)	**0.003**
IVSd, mm	13 (12–14)	12 (11–13)	13.5 (13–15)	**<0.001**
PWd, mm	11.5 (10–12)	11 (10–12)	12 (11–13)	**<0.001**
LVdd, mm	51.2 ± 5.4	49.4 ± 4.2	53.6 ± 5.9	**<0.001**
LVsd, mm	32.7 ± 5.9	30.5 ± 4.5	35.7 ± 6.3	**<0.001**
LV mass, g	130.2 ± 30.7	116.4 ± 20.7	148.3 ± 32.4	**<0.001**
E/A	1.1 ± 0.4	1.2 ± 0.4	1.1 ± 0.5	0.132
E/e’ septal	16.4 (12.7–20.9)	15 (11.6–18.3)	17 (13.4–25)	**0.011**
E/e’ lateral	13 (10.3–17)	12.5 (9.7–17.6)	13.4 (10.4–16.5)	0.388
E/e’ mean	14.4 (11.6–18.3)	14 (10.8–18.3)	15 (12.2–19)	0.107
LA volume index, mL/m^2^	47.3 (40.6–55.3)	43.5 (38.3–53)	51.4 (44.8–56.9)	**0.004**
Estimated PASP, mm Hg	33 (29–43)	33 (29.3–39.5)	35 (29–65)	0.272
GLS, % *	−18 ± 5	−20.1 ± 3.8	−15.2 ± 4.9	**<0.001**
CMR data				
IVSd, mm	13.3 ± 2	12.7 ± 1.9	14.2 ± 1.9	**<0.001**
PWd, mm	10.5 ± 1.9	9.8 ± 1.7	11.5 ± 1.8	**<0.001**
LVdd, mm	50.4 ± 6.1	48.8 ± 5.2	52.4 ± 6.7	**0.008**
LVsd, mm	33.7 ± 8.1	30.9 ± 6.6	37.2 ± 8.6	**<0.001**
LVEDV index, mL/m^2^	70.6 (61.5–80.6)	63.1 (54.9–74.6)	78.9 (70.4–99.8)	**<0.001**
LVESV index, mL/m^2^	20.6 (14.9–30.8)	16 (12.9–21.7)	29.8 (18.4–45.3)	**<0.001**
LVEF, %	66.7 ± 12.8	71.5 ± 7.7	60.6 ± 15.4	**<0.001**
LVEF < 50%, n (%)	9 (10.8)	1 (2.1)	8 (22.2)	**0.009**
LV mass, g	189.9 ± 68.1	152.3 ± 45.1	237.9 ± 62.2	**<0.001**
LV mass index, g/m^2^	92.5 (76.8–119.3)	79.3 (61.8–90.9)	119.8 (109.7–137.3)	**<0.001**
LGE prevalence	61 (73.5)	31 (66)	30 (83.3)	0.075
Native T1, ms ^#^	959.6 ± 33.7	946.5 ± 28.2	974.8 ± 33.6	**<0.001**
ECV, % ^#^	23.1 (20.8–24.9)	23 (20.7–24.9)	23.3 (21.2–25.2)	0.821
Histology data (*n* = 71)				
CVF total, % ^&^	16.1 ± 9.4	15.7 ± 8.7	16.6 ± 10.2	0.679
CVF midmyocardial, % ^&^	7 (3.8–11.9)	5.9 (3.6–9.1)	8.8 (4–12.6)	0.155
CVF subendocardial, % ^&^	21.6 ± 12.3	21.4 ± 10.9	21.8 ± 13.8	0.872

The boldface values indicate statistical significance. Continuous variables are presented as mean ± SD or median (IQR). Categorical variables are expressed as n (%). AV, aortic valve; AVA, aortic valve area; E, peak early velocity of the transmitral flow; CMR, cardiovascular magnetic resonance; CVF, collagen volume fraction; e’, peak early diastolic velocity of the mitral annulus displacement; GLS, global longitudinal strain; ECV, extracellular volume; IVSd, interventricular septum diastolic diameter; LVEDV, left ventricular end-diastolic volume; LVESV, left ventricular end-systolic volume; LVEF, left ventricular ejection fraction; LA, left atrium; LGE, late gadolinium enhancement; PASP, pulmonary artery systolic pressure measured by echocardiography; * value based on the data analysis in 77 patients; ^#^ values based on the data analysis in 67 patients; ^&^ values based on the data analysis in 70 patients.

**Table 3:** The univariate and multivariate regression analysis to identify prognostic factors for increased LV mass index and elevated native T1.

Variable	LV Mass Index				Native T1			
	Univariate Analysis		Multivariate Analysis		Univariate Analysis		Multivariate Analysis	
	OR (95% CI)	*p*-Value	OR (95% CI)	*p*-Value	OR (95% CI)	*p*-Value	OR (95% CI)	*p*-Value
Male sex	**3.67** **(1.39–9.69)**	**0.009**	2.91 (0.89–9.56)	0.078	1.07 (0.33–3.45)	0.914	-	-
Age, yrs	0.95 (0.90–1.00)	0.053	-	-	0.96 (0.90–1.03)	0.268	-	-
Hs-Tn-I, pg/L	1.00 (0.99–1.00)	0.497	-	-	1.00 (0.99–1.00)	0.697	-	-
BNP, pg/L	**1.00** **(1.00–1.01)**	**0.009**	1.00 (1.00–1.01)	0.309	1.00 (1.00–1.00)	0.600	-	-
LVEF, %	**0.91** **(0.86–0.97)**	**0.001**	0.94 (0.87–1.02)	0.110	**0.945** **(0.90–0.99)**	**0.016**	1.00 (0.92–1.08)	0.931
GLS, %	**0.73** **(0.62–0.87)**	**<0.001**	-	-	**0.86** **(0.75–0.99)**	**0.036**	0.93 (0.74–1.17)	0.546
QRS voltage	**1.09** **(1.03–1.15)**	**0.002**	0.98 (0.91–1.06)	0.679	**1.10** **(1.03–1.18)**	**0.006**	1.08 (0.99–1.18)	0.093
PQ duration, ms	1.01 (0.99–1.03)	0.262	-	-	1.00 (0.98–1.02)	0.885	-	-
QRS duration, ms	1.02 (0.99–1.05)	0.296	-	-	1.03 (0.99–1.07)	0.129	-	-
ECG strain	**12.89** **(3.90–42.55)**	**<0.001**	**7.10** **(1.46–34.48)**	**0.015**	**4.40** **(1.23–15.72)**	**0.023**	1.34 (0.26–7.03)	0.726

The boldface values indicate statistical significance. CI confidence interval, OR odds ratio. Abbreviations as in Table 1 and Table 2.

**Table 4:** ECG parameters before and at 3 and 12 months after surgical AVR.

Variables	Baseline (*n* = 83)	3-Month Follow-Up (*n* = 76)	12-Month Follow-Up (*n* = 59)
PQ duration, ms	165 (153.5–180)	164 (145.5–184)	163 (144.5–191.5)
QRS duration, ms	94 (86–102)	98.5 (88–115.5) *	96 (86–108)
S-L, mm	30 (23–39)	23 (18.5–27) *	19.5 (16–24) *
ECG strain, n (%)	36 (43.4)	26 (34.2)	10 (16.9) *

* *p* < 0.05 vs. baseline. Continuous variables are presented as mean ± SD or median (IQR). Categorical variables are expressed as n (%). Abbreviations as in Table 1.

**Table 5:** The comparison of baseline cardiovascular imaging and histology data of the study cohort, stratified by the presence of ECG strain at 1 year following AVR.

Variable	Patients without Persistent ECG Strain (*n* = 73)	Patients with Persistent ECG Strain (*n* = 10)	*p*-Value
Blood Tests			
eGFR, ml/min/1.73 m^2^	86 (73.5–90)	67.5 (60.3–80.8)	**0.019**
Hs-Tn-I, pg/L	9 (5–16)	17 (11.3–36.3)	0.065
BNP, pg/L	118.8 (60.7–285)	772.4 (148.3–1128.9)	**0.005**
Echocardiography data			
AVA, cm^2^	0.9 ± 0.2	0.7 ± 0.1	0.069
AVA index, cm^2^/m^2^	0.45 ± 0.1	0.40 ± 0.1	0.220
Peak AV velocity, m/s	4.9 ± 0.6	4.6 ± 0.6	0.122
Mean AV gradient, mm Hg	59.2 ± 17.4	52.6 ± 13.4	0.249
IVSd, mm	13 (12–15)	13 (11–14.3)	0.949
PWd, mm	11 (10–12)	12 (10.5–13.5)	0.553
LVdd, mm	50 (47–53)	56.5 (51.3–58)	**0.014**
LVsd, mm	31.8 ± 5	39.1 ± 8.3	**<0.001**
LV mass, g	127.7 ± 29.6	153.4 ± 32.1	**0.023**
E/A	1.2 ± 0.4	1 ± 0.6	0.299
E/e’ septal	16.3 (12.7–20.4)	16.7 (11.7–23.2)	0.994
E/e’ lateral	13.3 (10.4–17.1)	10.8 (9–22.2)	0.558
E/e’ mean	14.5 (11.8–18.3)	12.7 (11.1–22)	0.716
LA volume index, mL/m^2^	46.2 (29.6–55)	54.5 (48.6–56.8)	0.118
Estimated PASP, mm Hg	33 (29.5–40.5)	54 (26–70)	0.250
GLS, % *	18.6 ± 4.4	12.8 ± 6.7	**<0.001**
CMR data			
IVSd, mm	13.3 ± 2.1	13.6 ± 1.9	0.648
PWd, mm	10 (9–12)	10 (9.5–11.4)	0.861
LVdd, mm	49 (46–52.5)	55.5 (50.8–58.8)	**0.013**
LVsd, mm	32.6 ± 7.5	41.4 ± 8.8	**<0.001**
LVEDV index, mL/m^2^	70.5 (61.1–78.2)	99.2 (63.7–129)	**0.010**
LVESV index, mL/m^2^	18.7 (14.7–28.5)	52.7 (18.9–83.8)	**0.003**
LVEF, %	68.9 ± 10.3	51 ± 18.2	**<0.001**
LVEF < 50%, n (%)	4 (5.5)	5 (50)	**<0.001**
LV mass, g	167 (138.3–239)	236 (185.8–255)	0.072
LV mass index, g/m^2^	86.4 (75.5–118.5)	119.8 (101.5–131.9)	0.052
RVEDV, mL	124.3 ± 31.5	132.5 ± 28.6	0.440
RVESV, mL	43.5 (35–58.6)	57.7 (50.2–71.5)	**0.020**
RVEF, %	62.1 ± 7.5	52.5 ± 16.3	**0.002**
LGE prevalence	53 (72.6)	8 (80)	1.000
Native T1, ms ^#^	955 (934.5–976)	965 (943–1004.3)	0.374
ECV, % ^#^	22.6 ± 3.7	23.7 ± 2.6	0.401
Histology data (*n* = 71)			
CVF total, % ^&^	15.6 ± 8.4	19 ± 14.2	0.293
CVF midmyocardial, % ^&^	7.3 ± 4.7	12.5 ± 9.9	**0.009**
CVF subendocardial, % ^&^	21.4 ± 11.5	22.6 ± 16.6	0.770

The boldface values indicate statistical significance. Continuous variables are presented as mean ± SD or median (IQR). Categorical variables are expressed as n (%). Abbreviations as in Table 1 and Table 2.

## Data Availability

The datasets are available upon request to the corresponding author.

## Abbreviations

AS	aortic stenosis
AVA	aortic valve area
AV	aortic valve
AVR	aortic valve replacement
BNP	brain natriuretic peptide
CAD	coronary artery disease
CMR	cardiovascular magnetic resonance
CVF	collagen volume fraction
ECG	electrocardiography
ECV	extracellular volume
GLS	global longitudinal strain
Hs-Tn-I	high-sensitivity troponin I
LGE	late gadolinium enhancement
LV	left ventricle
LVEF	left ventricular ejection fraction
TAVI	transcatheter aortic valve implantation
